# Multiple liver insults synergize to accelerate experimental hepatocellular carcinoma

**DOI:** 10.1038/s41598-018-28486-8

**Published:** 2018-07-06

**Authors:** James M. Henderson, Natasa Polak, Jinbiao Chen, Ben Roediger, Wolfgang Weninger, James G. Kench, Geoffrey W. McCaughan, Hui Emma Zhang, Mark D. Gorrell

**Affiliations:** 1 Centenary Institute, The University of Sydney, Newtown, New South Wales, 2042 Australia; 2 The University of Sydney Faculty of Medicine and Health, New South Wales, 2006 Australia; 3 Royal Prince Alfred Hospital, Camperdown, New South Wales, 2050 Australia; 4 Charles Perkins Centre, The University of Sydney, New South Wales, 2006, Australia

## Abstract

The urgent unmet need for hepatocellular carcinoma (HCC) therapies is addressed here by characterising a novel mouse model of HCC in the context of ongoing liver damage and overnutrition. Male C57Bl/6J mice were treated with diethylnitrosamine (DEN) and thioacetamide (TAA), and some were provided with an atherogenic high fat diet (HFD). Inflammation, steatosis, fibrosis, 87 genes, liver lesions and intratumoural leukocyte subsets were quantified up to 24 weeks of age. Adding HFD to DEN/TAA increased fibrosis, steatosis and inflammation, and the incidence of both HCC and non-HCC dysplastic lesions. All lesions contained α-SMA positive fibroblasts. Macrophage marker F4/80 was not significantly different between treatment groups, but the macrophage-associated genes *Arg-1* and *Cd47* were differentially expressed. Fibrosis, cancer and cell death associated genes were upregulated in DEN/TAA/HFD livers. Fewer Kupffer cells and plasmacytoid dendritic cells were in tumours compared to control liver. In conclusion, combining a hepatotoxin with an atherogenic diet produced more intrahepatic tumours, dysplastic lesions and fibrosis compared to hepatotoxin alone. This new HCC model provides a relatively rapid means of examining primary HCC and potential therapies in the context of multiple hepatotoxins including those derived from overnutrition.

## Introduction

Hepatocellular carcinoma (HCC) is the fifth most commonly diagnosed cancer in men and is the second leading cause of cancer death worldwide. With approximately 850,000 new cases diagnosed worldwide every year, HCC is the leading cause of cirrhosis-based fatality^1,2^. There is an urgent unmet need for effective therapies for HCC. Current mouse models of HCC are generally limited by the time taken to carcinogenesis, typically 8–12 months^3,4^. Faster models and models reflecting the variety and multiplicity of causes seen in patients may facilitate research into novel therapeutics.

HCC arises from liver cirrhosis, a pathological change in the structure and function of the liver in response to insults and injury, resulting in inflammation and fibrosis. Common causes of liver cirrhosis include hepatitis C virus, hepatitis B virus, as well as alcohol-related cirrhosis and non-alcoholic fatty liver disease (NAFLD) associated with obesity^1,2^. NAFLD is the most common chronic liver disease in developed countries and has been identified as a hepatic manifestation of metabolic syndrome^5^. NAFLD incorporates a wide range of conditions from simple steatosis to non-alcoholic steatohepatitis (NASH), which accompanies inflammation, necrosis and fibrosis and is increasingly associated with HCC^6^. Obesity is an independent risk factor for the development of HCC, conferring increased probabilities of liver cancer of 4.2% in men and 2.8% in women^7,8^.

The three popular configurations of mouse models of HCC are xenografts, genetically-engineered mice and chemically-induced cancers^4^. Genetic models of HCC are suitable for investigating specific genes, but limitations include the potential for effects during embryogenesis, and activation of compensatory pathways. Models involving wild-type mouse strains are multigenic and so may more broadly reflect natural situations.

Developing effective therapies for HCC is hindered by limitations inherent in animal models of HCC, particularly the length of time taken to induce carcinogenesis and the inability to completely recapitulate the human tissue microenvironment^4^. N-nitrosodiethylamine (diethylnitrosamine; DEN) and thioacetamide (TAA) are two of the best-characterized hepatotoxins used in HCC models, and are widely used to induce proliferative and neoplastic changes within mouse liver. The time to induce carcinogenesis in the DEN/TAA model is generally no shorter than 32 weeks.

Here, we examined the incorporation of an atherogenic high fat diet (HFD) into a DEN/TAA model of HCC. We characterized the histopathological and molecular features of our DEN/TAA/HFD model, particularly in relation to dysplastic lesions, tumours, fibrosis and inflammation. Compared with the DEN/TAA model of HCC, mice in the DEN/TAA/HFD model contained more intrahepatic fibrosis, inflammation and HCC.

## Results

### Model of chronic liver injury

**Figure 1:**
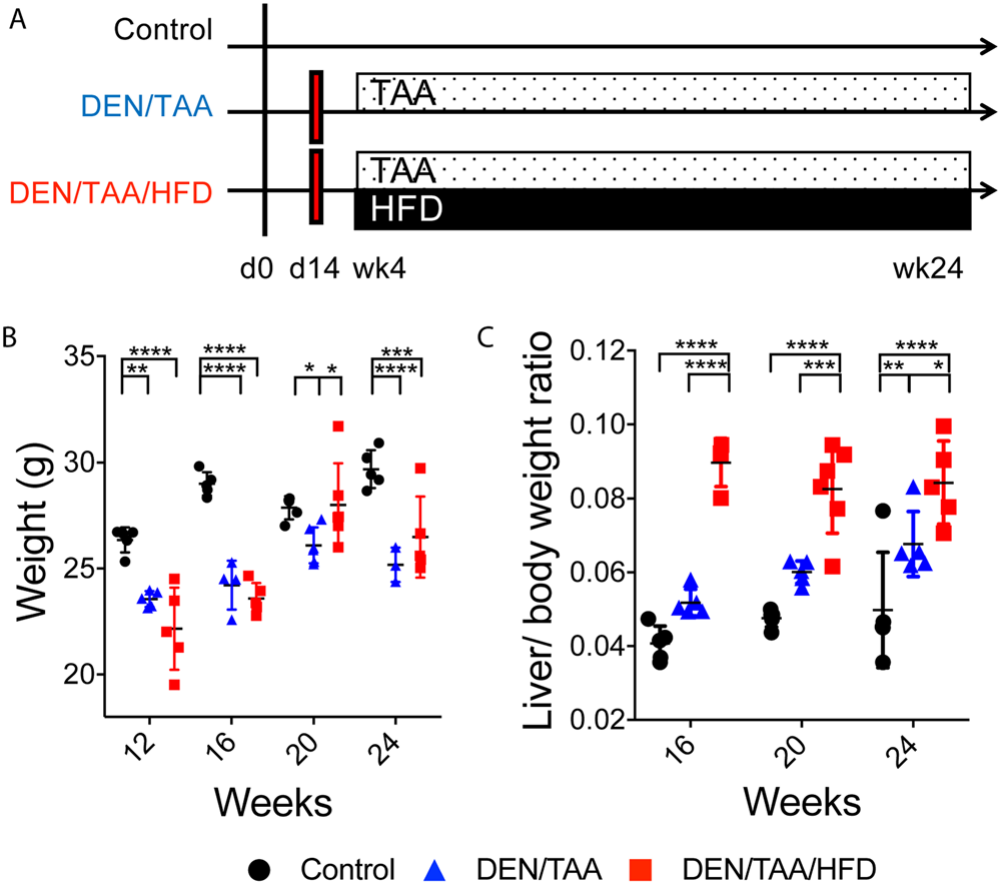
Model of chronic liver injury. **(A)** Overview of treatments: N-nitrosodiethylamine (DEN; d14)/Thioacetamide (TAA; wk4–24)/High fat diet (HFD; wk4–24), DEN/TAA, and control. **(B**,**C)** Mouse body weight at death and liver to body weight ratio. n = 5–6. Mean and SEM. Statistical analysis used Two-way ANOVA with Tukey’s multiple comparison test, *p < 0.05, **p < 0.01, ***p < 0.001, ****p < 0.0001 compared to age-matched control.

DEN is a potent and reproducible inducer of hepatocellular carcinogenesis and injection with a single dose at 14 days of age optimizes intrahepatic DNA damage^4,9^ (Fig. 1A). TAA is a potent inducer of liver injury and fibrosis, which facilitates premalignant transformations in hepatocytes. The atherogenic diet high in fat, cholesterol and sucrose (HFD) was expected to metabolically damage hepatocytes. Compared to saline-injected mice without TAA, all DEN/TAA-treated mice had decreased body weight throughout the following 5 months (Fig. 1B), as expected^10^. The addition of HFD did not compensate for this TAA induced decreased body weight (Fig. 1B). Nevertheless, liver weights were significantly increased in the HFD fed mice at 16, 20 and 24 weeks of age (Fig. 1C).

### Pathological changes in DEN/TAA/HFD-treated mice

**Figure 2:**
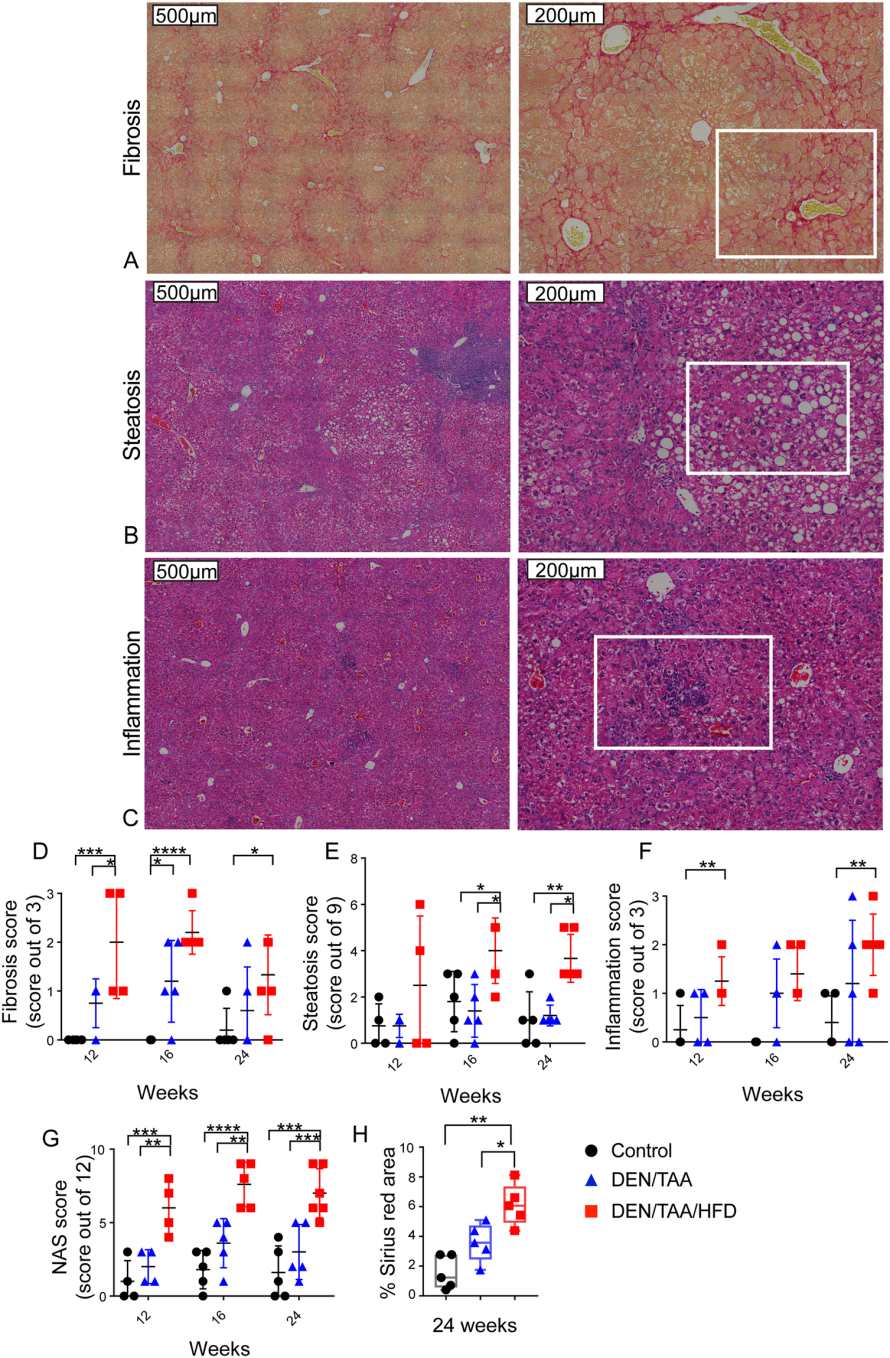
Pathological changes in liver. Picro-Sirius red **(A)** and H&E **(B**,**C)** stained liver paraffin sections from a mouse treated with DEN/TAA/HFD. Areas of fibrosis **(A)**, steatosis **(B)** and inflammatory cell aggregates **(C)** are boxed with white lines. Histological quantification of fibrosis (**D**), steatosis (**E**), inflammatory cell aggregates (**F**) and NAFLD activity scores (NAS) **(G)** were performed. Histological quantification of collagen from Sirius red staining of liver from 24-week-old mice (n = 5–6) treated with DEN/TAA/HFD **(H)**. Statistical analysis used two-way ANOVA with Tukey’s multiple comparison test (**D–G**) or Kruskal-Wallis test (**F**), *p < 0.05, **p < 0.01, ***p < 0.001, **** p < 0.0001. Scale Bars = 200 μm or 500 μm.

Histological analyses of the livers from these mice at 12, 16 and 24 weeks of age, revealed steatosis, fibrosis and inflammatory cell aggregates in DEN/TAA/HFD treated mice (Fig. 2A–C**)**. Most of the DEN/TAA treated mice exhibited fibrosis. However, the severity of fibrosis was greater in DEN/TAA/HFD mice (Fig. 2D,H). No statistically significant change in fibrosis between 24 and 16 weeks was detected in the DEN/TAA/HFD treated mice p = 0.1212 (Fig. 2D). Liver steatosis at weeks 16 and 24 was significantly increased in mice on HFD (Fig. 2E), in concordance with the liver weight data (Supporting Information 5). The DEN/TAA/HFD livers showed significant increases in the number of inflammatory cell aggregates at 12 and 24 weeks compared to untreated controls (Fig. 2F**)**. The addition of HFD to the DEN/TAA model showed a statistically significant increase of the NAFLD activity score at all time points measured (Fig. 2G). Together, these results indicate that our DEN/TAA/HFD protocol is sufficient to drive all three pathological changes within the liver, and that the HFD increases the inflammatory and fibrotic changes.

### Cancer burden in DEN/TAA/HFD-treated mice

**Figure 3:**
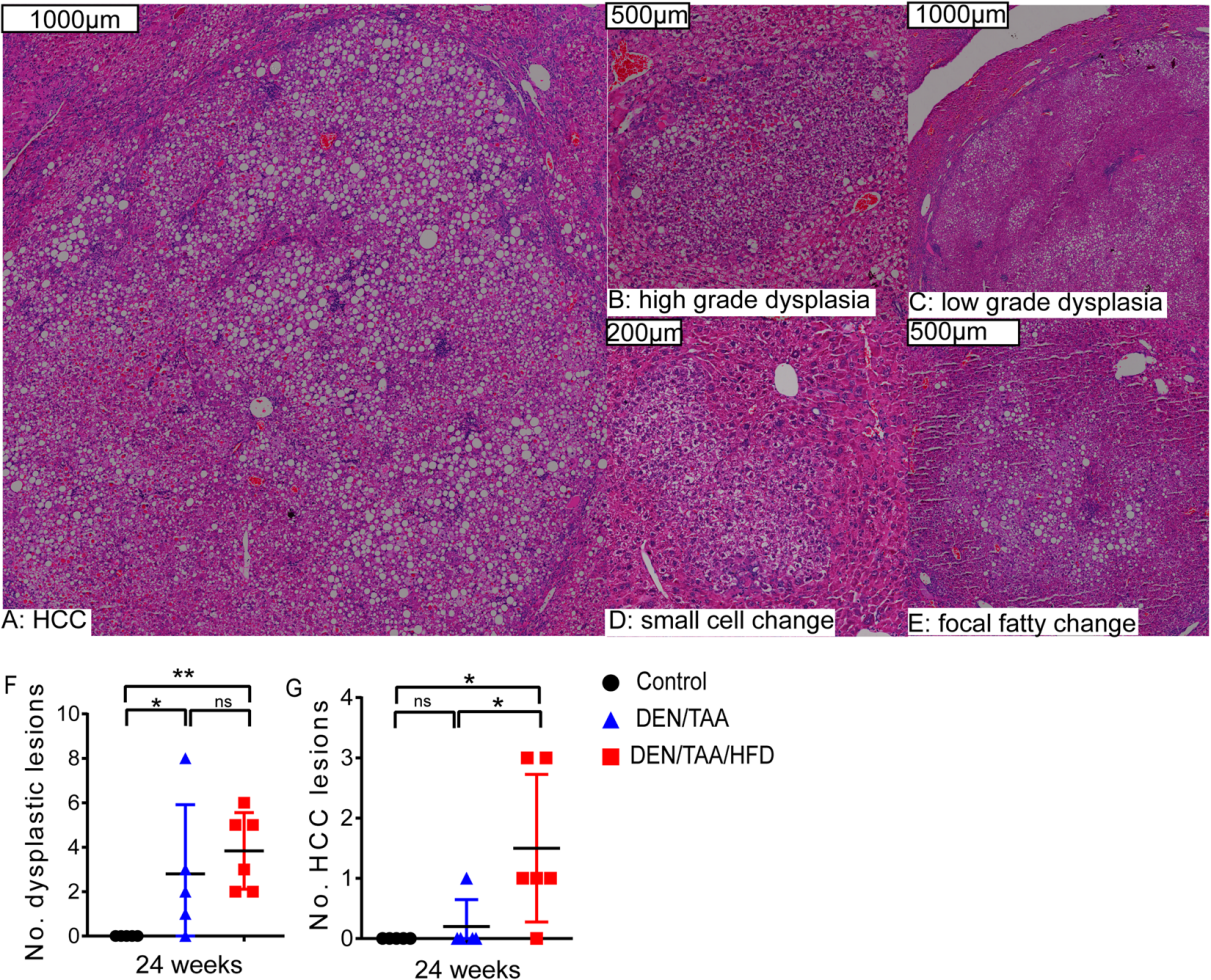
Burden of tumours and other lesions in liver. The types (**A–E**) and numbers of dysplastic (**F**) and HCC (**G**) lesions observed. n = 5–6. Scale Bars = 1000 μm (**A**,**C)**, 500 μm (**B**,**E**) and 200 μm (**D**). Statistical analysis used one-tailed Mann-Whitney U test, *p < 0.05, **p < 0.01.

Classical DEN/TAA models induce neoplastic changes in the liver at 32–40 weeks of age^3,4,11–13^. We were therefore interested in whether the HFD altered the quantity or appearance of dysplastic foci. We were able to identify and quantify lesions on haematoxylin and eosin (H&E) stained sections **(**Fig. 3A–E, Supporting information 6). Control livers from untreated mice contained no lesions (Supporting information 6). At 24 weeks of age, total numbers of low and high-grade dysplastic lesions in the DEN/TAA- and DEN/TAA/HFD-treated mice increased compared to control mice **(**Fig. 3F). The inclusion of HFD led to an increase in the number of histologically confirmed HCC lesions, compared to both the DEN/TAA and the control mice **(**Fig. 3G**)**. Collectively, these results suggest that the addition of HFD to the DEN/TAA protocol induced changes that increased the incidence of HCC and dysplasia at 24 weeks of age.

We were then interested in characterizing the pathological and molecular features of our DEN/TAA/HFD model, particularly in relation to the fibrotic and inflammatory changes, both of which were augmented by the addition of HFD (Fig. 2D,F).

### Fibrotic changes in DEN/TAA/HFD-treated mice

**Figure 4:**
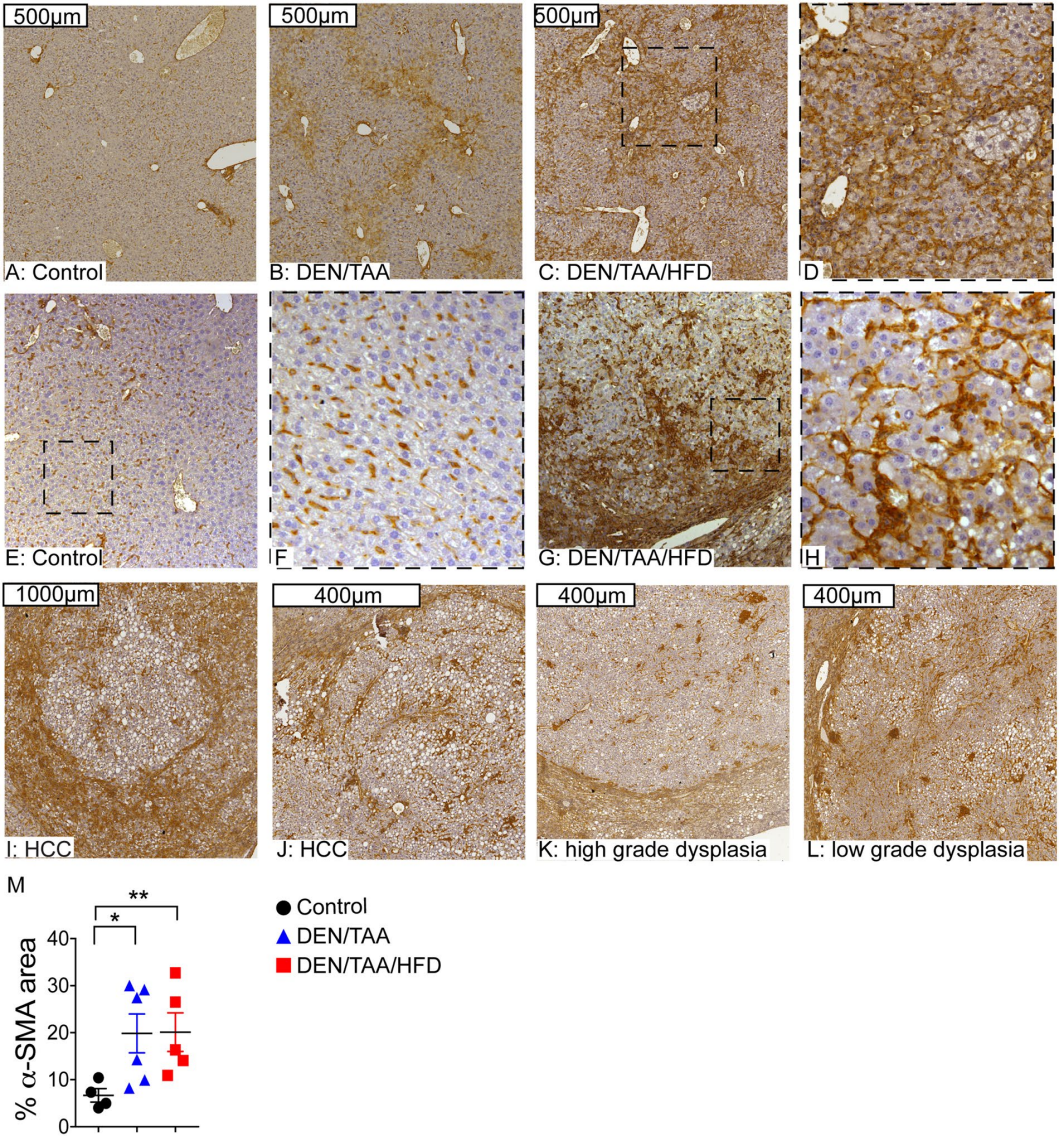
α-SMA immunohistochemistry in liver. **(A–D)** α-SMA immunostaining (brown) and haematoxylin counterstain in control **(A)**, DEN/TAA **(B)** and DEN/TAA/HFD **(C**,**D)** treatment groups. **(E–L)** Examples of the α-SMA expression in different types of lesions observed in DEN/TAA/HFD treated mice. Boxes formed by black dashes indicate higher magnification of adjacent images. **(M)** Quantitation of α-SMA immunopositivity by area in the whole liver section. Representative data from 5–6 mice per group. Statistical analysis used Kruskal-Wallis test, *p < 0.05, **p < 0.01. Scale Bars = 400 μm (**J–L**), 500 μm (**A–C**), and 1000 μm (**I**).

Hepatic fibrosis is a ubiquitous response to chronic injury of the liver, and potentiates malignant transformation^14^. It is widely accepted that liver fibrosis is predominantly driven by hepatic stellate cells, the resident perisinusoidal mesenchymal cell population^15^. Following liver insult, these cells undergo activation from a quiescent, vitamin A-storing cell to a proliferative, myofibroblast like, alpha smooth muscle actin (α-SMA)-expressing cell exhibiting upregulated collagen synthesis. In our model, α-SMA expression (measured as % positive area/total tissue area) increased in both the DEN/TAA/HFD and DEN/TAA livers compared to control (Fig. 4A–D,M). Furthermore, we found consistent infiltration of α-SMA-positive fibroblasts into all lesions observed, regardless of the level of transformation (Fig. 4E–L).

### Quantitative assessment of intrahepatic gene expression

**Figure 5:**
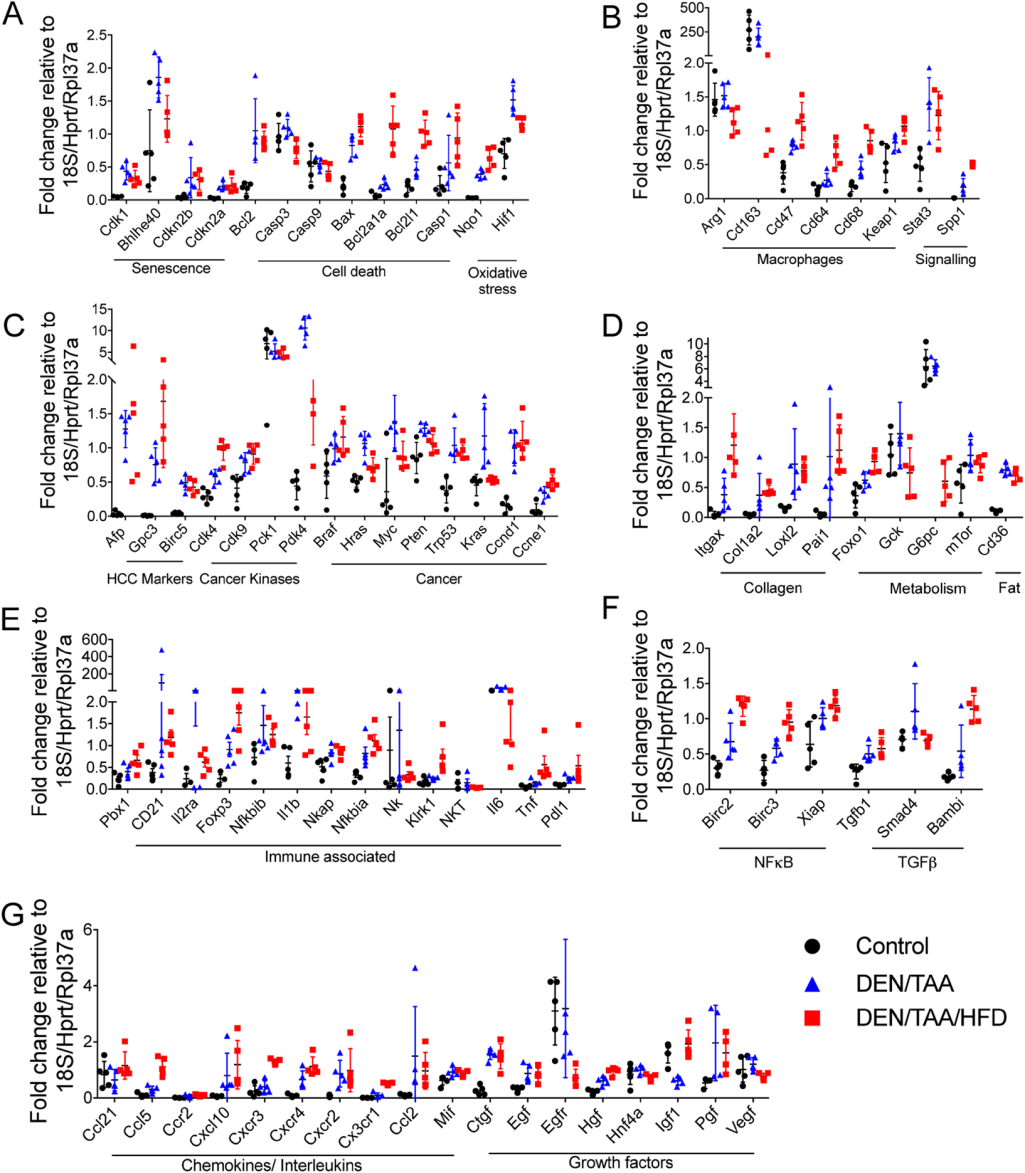
Gene expression in liver. Gene expression normalized to housekeepers *18S*, *Hprt* and *Rpl37a* of individual mice plotted with mean and standard deviation (n = 5 mice per group). Statistical analyses used one-tailed Mann-Whitney U test. Statistical significance is shown in Table 1.

Gene expression was investigated in the livers of 24-week old mice. qRT-PCR was performed on 87 genes **(**Fig. 5, Table 1**)**, which were selected based on their established involvement in cancer, metabolism, fibrosis and inflammation pathways (Supporting information 4**)**.

**Table 1:** Quantitative PCR by TaqMan array card on liver mRNA obtained at 24 weeks of age.

Gene name	Significance			Gene name	Significance			Gene name	Significance		
	DTH v Swc	DTH v DTC	DTC v Swc		DTH v Swc	DTH v DTC	DTC v Swc		DTH v Swc	DTH v DTC	DTC v Swc
Afp	**		**	Birc3	**	**	*	Ctgf	**		**
Gpc3	**		**	Xiap	*			Egf	**		**
Birc5	**		**	Tgfb1	**	**		Egfr	**	**	
Cdk4	**	**	**	Smad4		**	*	Hgf	**	*	**
Cdk9	**		**	Bambi	**		**	Hnf4a		*	
Pck1				Pbx1	*			Igf1		**	**
Pdk4	**	**	**	Cd21	**			Pgf			
Braf	*		*	Il2ra				Vegf		**	
Hras	*	*	**	Foxp3	*		*	Itgax	**	*	*
Myc		*	*	Nfkbib	*			Col1a2	**		**
Pten			**	Il1b			**	Loxl2	**		**
Trp53	**		**	Nkap	*			Pai1	**		**
Kras		**	**	Nfkbia	**		**	Foxo1	**	**	**
Ccnd1	**		**	Nk				Gck			
Ccne1	**	*	*	Klrk1	**	**		G6pc	**	**	
Cdk1	**		**	NKT				mTor			*
Bhlhe40				Il6		**	*	Cd36	**		**
Cdkn2b	*		*	Tnf	**	**	**	Arg1		**	
Cdkn2a	**		**	Pdl1	**		**	Cd163	**	**	
Bcl2	**		**	Ccl21				Cd47	**		**
Casp3		**		Ccl5	**	**	*	Cd64	**	*	*
Casp9				Ccr2	**		**	Cd68	**	**	**
Bax	**		**	Cxcl10	**		**	Keap1	**		
Bcl2a1a	**	**	**	Cxcr3	**	**		Stat3	*		**
Bcl2l1	**	**	**	Cxcr4	**		**	Spp1	**	**	**
Casp1	**			Cxcr2	*		*				
Nqo1	**	*	**	Cx3cr1	**	**	**				
Hif1	**	**	**	Ccl2	**		**				
Birc2	**	*	*	Mif	**		**				

DTH = DEN/TAA/HFD, DTC = DEN/TAA/Chow, Swc = control. Statistical analysis used one-tailed Mann-Whitney test; *p < *0*.*05*, **p < *0*.*01*. Full gene names and probes are listed in Supporting information 4.

A number of cell death associated genes (*Bax*, *Bcl2a1a*, *Bcl2l1*, *Casp1*, *Birc2*, *Birc3*, *Birc5 and Xiap*) were upregulated in DEN/TAA/HFD livers compared to control, and some of these were also upregulated in DEN/TAA/HFD compared to DEN/TAA (Fig. 5A,F; Table 1). Caspase-1 is the hallmark of canonical pyroptosis^16,17^, suggesting that there may be ongoing intrahepatic pyroptosis in DEN/TAA/HFD treated mice. Several growth factor genes were upregulated in DEN/TAA/HFD liver compared to control (Fig. 5G; Table 1).

Upregulation of the cancer associated genes *Trp53*, *Ccnd1*, *Cdk4*, *Cdk9*, *Pdk4*, *Ccne1 and Spp1*, and the senescence associated gene *Cdk1* occurred in DEN-treated mice compared to control mice. The HCC-associated genes *Afp*, *Gpc3*, *Birc5*, *and Braf*, and oxidative stress genes *Nqo1* and *Hif1* were significantly upregulated in DEN-treated mice compared to control mice. The cancer-associated genes *Hras*, *Myc*, *Pten and Kras* were significantly upregulated in DEN/TAA treated mice, and trended towards significance in the DEN/TAA/HFD mice, compared to control (Fig. 5C; Table 1**)**.

DEN/TAA/HFD treated mice upregulated the fatty acid transporter *Cd36* and all collagen- and fibrosis-associated genes examined (*Itgax*, *Col1a2*, *Loxl2*, *Pai1*, *Tgfb1*, *and Ctgf*) when compared to control mice (Fig. 5D,F,G; Table 1). Most of the fibrosis-associated genes were also upregulated in the DEN/TAA treated mice compared to control mice. The upregulation of many key immune associated genes (*Pbx1*, *Cd21*, *Foxp3*, *Nkap*, *Nfkbib*, *Nfkbia*, *Klrk1*, *Tnf*, *Pdl1*, *Ccl2*, *Ccl5*, *Ccr2*, *Cxcr3*, *Cx3cr1 and Mif)* was seen in all DEN/TAA/HFD treated mice when compared to control mice, and many of these genes were also upregulated in DEN/TAA treated mice (Fig. 5E; Table 1**)**. These data suggest that signals for inflammation and fibrosis were stronger in the DEN/TAA/HFD treated than DEN/TAA treated mice.

### Leukocyte infiltration

**Figure 6:**
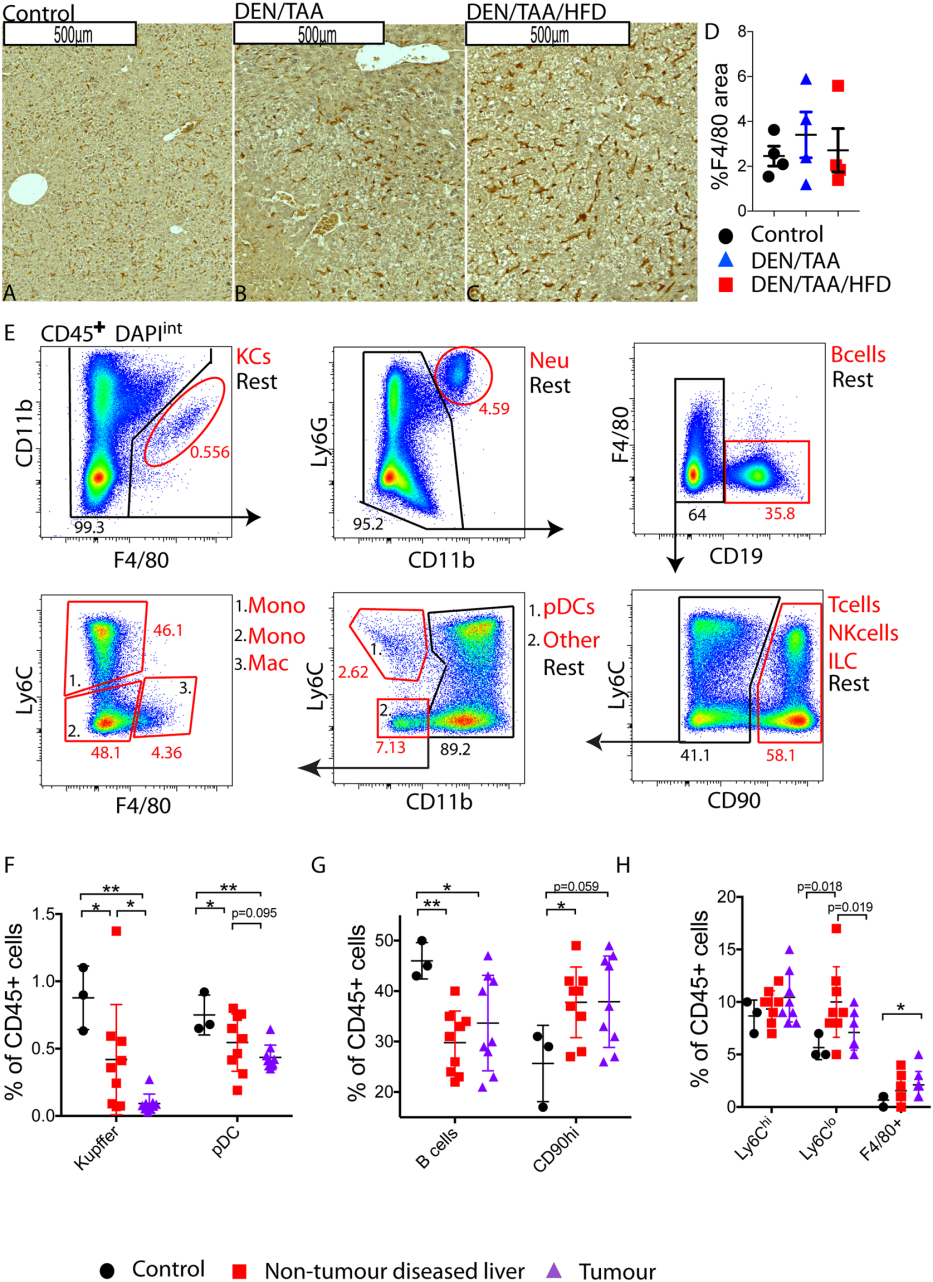
Leukocyte infiltration into HCC in the DEN/TAA/HFD model. **(A–C)** F4/80 immunostaining (brown) of liver sections, n = 5–6. Scale Bars = 500 μm. **(D)** Quantitation of F4/80 immunostains (n = 4 mice per group). **(E)** Clockwise gating strategy from CD45^+^ live cells, showing putative immune cell populations (sub-population number as % of parent population shown in red, remaining cells shown in black). **(F–H)** Flow cytometry analysis of immune cell sub-populations (n = 3–12 mice per group). KC; Kupffer cell, Neu; neutrophil, Mono; monocyte, pDC; plasmacytoid dendritic cell, NK; natural killer, ILC; innate lymphoid cell, Mac; non-Kupffer macrophages Ly6C^hi^; Ly6C^hi^ monocyte, Ly6C^lo^; Ly6C^lo^ monocyte. Statistical analysis used Kruskal-Wallis test **(D)**, or one-tailed Mann-Whitney test **(F–H)**, *p < 0.05, **p < 0.01.

Inflammatory cell aggregates were observed in our model (Fig. 2F), so we further examined leukocyte infiltration. Immunostaining with the macrophage marker F4/80 was not significantly different between treatment groups at 24 weeks of age (Fig. 6A–D**)**. However, significant changes in expression levels of the macrophage-associated genes *Cd163*, *Cd47*, *Cd64*, *Cd68 and Keap1* indicated a change in macrophage phenotype between all treatment groups (Fig. 5B).

We also examined immune populations in tumours compared to non-tumour diseased tissue of cancer-bearing livers and control livers by flow cytometry. To achieve this, we used 36-week-old DEN/TAA/HFD mice, in which many lesions were sufficiently large to be reliably micro dissected from the surrounding (diseased) liver. CD45^+^ immune cells were then gated into subpopulations; Kupffer cells (CD11b^+^F4/80^hi^), neutrophils (Ly6G^hi^CD11b^hi^), B cells (CD19^hi^), T cells, natural killer cells and innate lymphoid cells (CD90^hi^), plasmacytoid dendritic cells (CD11b^–^Ly6C^hi^), monocytes (CD11b^hi^Ly6C^hi/−^), and non-Kupffer macrophages (CD11b^+^F4/80^int^) (Fig. 6E). DEN/TAA/HFD tumours showed decreased Kupffer and plasmacytoid dendritic cell populations compared to control livers (Fig. 6F). The non-tumour area of cancer-bearing liver showed decreased B cell (Fig. 6G) and increased non-Kupffer macrophage (Fig. 6H) populations compared to control liver. When comparing tumour and non-tumour diseased tissue, no significant differences in immune populations were seen.

### Cancer markers

**Figure 7:**
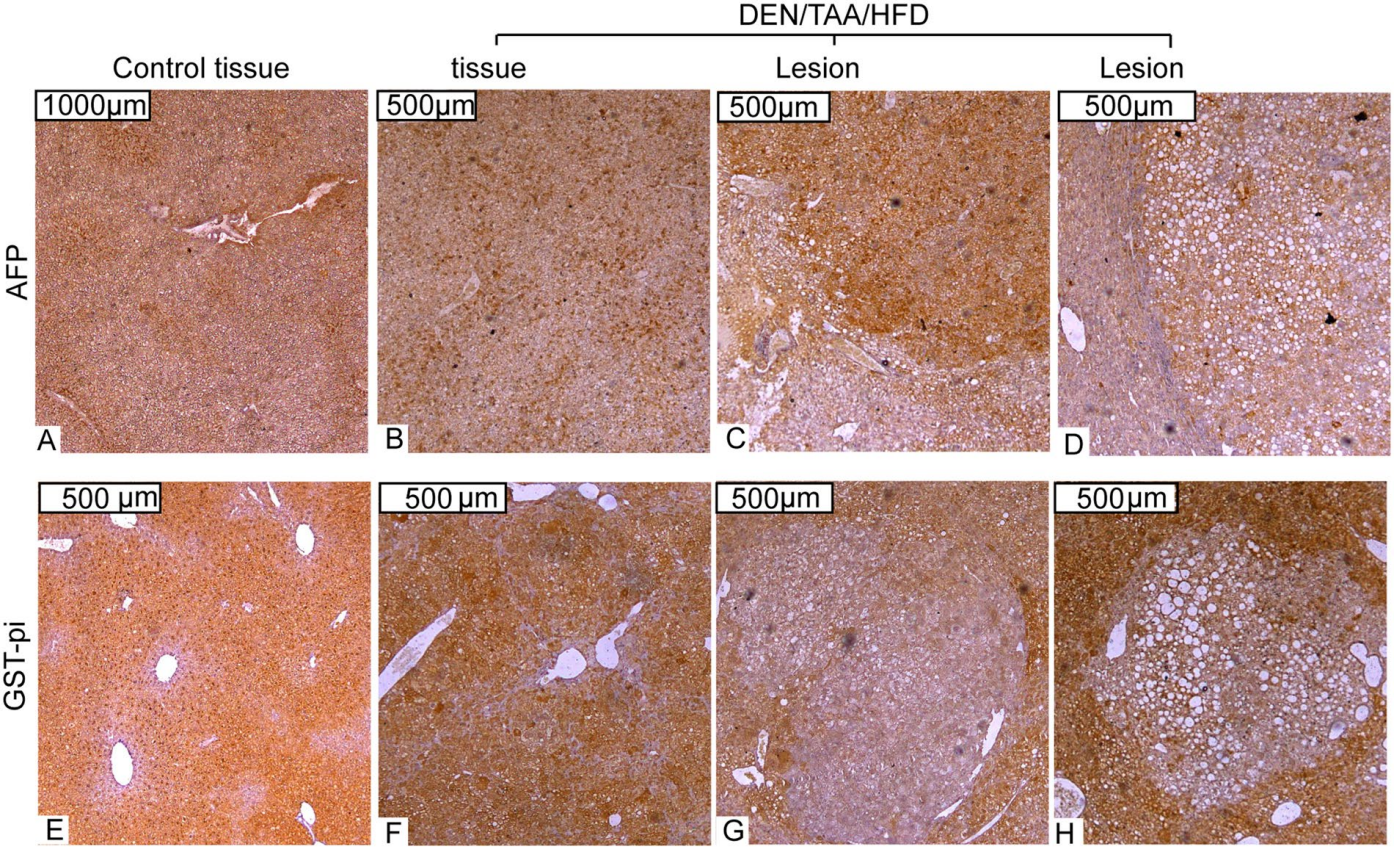
Cancer Biomarkers. Alpha fetoprotein (AFP) **(A–D)**, and glutathione S-transferase Pi (GST-pi) **(E–H)** immunostaining (brown) of liver sections of control and DEN/TAA/HFD treated mice. Lesions were identified by encapsulation, increased steatosis, and changes in hepatocyte architecture. Representative data from 4 mice per group. Scale Bars = 500 μm (**B–H**), 1000 μm (**A**).

Immunostains of Alpha fetoprotein (AFP) and glutathione S-transferase Pi (GST-pi) in lesions showed limited numbers of lesions immunopositive for AFP (4/11) or GST-pi (3/11) (Fig. 7).

## Discussion

The addition of HFD to the DEN/TAA led to an increased number of confirmed HCC lesions at 24 weeks of age. Significant fibrosis responses were seen at the mRNA, protein and macroscopic level. Increased inflammation was also observed by H&E stain, accompanied by the upregulation of several immune associated genes. Together this shows the addition of a HFD increased carcinogenesis in mouse livers when compared to DEN/TAA treatment and did so in the context of liver inflammation, steatosis and fibrosis.

The multiple-hit hypothesis of the pathogenesis of NASH^18^ is that multiple hepatotoxic insults occurring in parallel, rather than consecutively, result in NASH and subsequent HCC development. Our DEN/TAA/HFD model fits with epidemiological studies indicating that obesity is the largest risk for liver cancer^7^. The addition of HFD to a DEN model of HCC has been shown to promote tumourigenicity^12^, and involves low-grade inflammation. A DEN/HFD comparator was not included in the present study because we have shown that our HFD does not cause liver fibrosis^19^ and others have shown that DEN/HFD requires 8 months to produce consistent HCC. In contrast, a different diet, a choline-deficient HFD, with a single DEN injection can induce HCC from 24 weeks of age, despite causing little fibrosis^20^.

An additional advantage of our DEN/TAA/HFD model is tolerability for the animals. Some models employ additional doses of DEN, but such a regime can cause mortality for the animals without hastening the onset of HCC^21^.

Chronic liver injury is associated with the activation and transdifferentiation of myofibroblasts and hepatic stellate cells that display increased proliferation, contractility, fibrogenesis, matrix degradation, and chemotaxis and cytokine release. Activated stromal fibroblasts are considered a key component of solid tumours, with roles in tumour progression, growth and metastasis at all stages^22–24^. In our DEN/TAA/HFD model, α-SMA expression significantly increased, indicating that hepatic stellate cells were activated and had probably proliferated. The upregulation of collagen- and of fibrosis-associated genes indicated increased extracellular matrix deposition in DEN/TAA/HFD mice compared to DEN/TAA and control. *Ctgf* and *Tgfb1*, which promote intrahepatic tissue remodelling and fibrosis, were prominent.

Gene expression patterns seen in human HCC with poor prognosis are similar to DEN-induced tumours in mouse^25^. The gene signature of DEN/TAA/HFD treated mice is crucial to understand the mechanisms of tumour progression. Here we showed that in the DEN/TAA/HFD model upregulated genes were associated with cell death, cell proliferation, cell signaling, cancer, fibrosis, metabolism, and the immune system **(**Table 1**)**. These gene changes, including *Trp53*, *Ccnd1*, *Ccne1*, *Cdkn2a* and *Pdl1*, are in line with previous studies of HCC models and human HCC^4,5,26,27^ and would be expected to mediate synergistic liver damage and tumour insults. These gene expression changes, particularly by the inhibitors of apoptosis *Birc2*, *Birc3*, *Birc5* and *Xiap*, were more pronounced in DEN/TAA/HFD treated than in DEN/TAA treated mice.

Liver contains several distinct subsets of macrophages and dendritic cells. During HCC, Kupffer cells and macrophages clear premalignant senescent hepatocytes and produce tumorigenic signals during chronic inflammation^28^. Tumour-associated macrophages contribute to HCC progression by promoting tumour cell proliferation, neo angiogenesis, and metastasis^28^. Tumour-associated macrophages represent the main inflammatory cells associated with cancer-related inflammation, immune suppression, and angiogenesis^29^. Macrophages were present both inside and outside the DEN/TAA/HFD induced lesions, but there were no obvious histological differences between groups based on F4/80+ immunopositivity (Fig. 6D**)**. By flow cytometry, fewer Kupffer cells and more non-Kupffer macrophages were isolated from tumours compared to control livers (Fig. 6F,H).

Macrophage phenotypes vary and form a continuous spectrum, from classically activated macrophages that produce IL-12 and iNOS, to alternatively activated macrophages that produce IL-10 and arginase. The HCC microenvironment promotes the alternatively activated macrophage polarization^30^. Differential gene expression indicated differences in macrophage phenotype between treatment groups but the expression patterns did not align with a recognised macrophage subtype. Decreased expression of *Arg1* (encoding arginase) in the DEN/TAA/HFD treated mice compared to DEN/TAA treated mice indicates a shift away from the alternatively-activated macrophage phenotype **(**Table 1**)**. CD68 and CD163 positive macrophages have been characterized in HCC as being both classically and alternatively polarized macrophages^31^. CD47, which was upregulated in all DEN/TAA/HFD treated mice compared to DEN/TAA treated mice and controls, is a negative prognostic factor for a variety of cancers, and acts as a “don’t eat me” signal for many cancers^32^.

HCC is considered an inflammation-associated cancer^33^. DEN-induced HCC typically exhibits substantial leukocyte infiltration in mice, similar to human HCC^34,35^. T cell-deficient mice develop severe fibrosis, whereas B cell-deficient mice show attenuated fibrosis^28,36^. Moreover, NASH-driven HCC is accompanied by an accumulation of IgA^+^ B cells that suppress cytotoxic T-cells in anti-tumour immune responses^37^. Concordantly, we observed more B cells (CD45^+^CD19^hi^) and numerous CD45^+^CD90^hi^ cells (T cells, natural killer cells and innate lymphoid cells) in both the tumour and non-tumour diseased tissue of cancer-bearing livers compared to healthy controls (Fig. 6F**)**. Thus, our DEN/TAA/HFD model produced extensive inflammation and fibrosis that recapitulates human HCC.

Traditional biomarkers for HCC in murine models include GST-pi and AFP, but both exhibit high levels of false negatives limiting their utility^27^. At the mRNA level, we saw upregulation of the HCC markers *Afp*, *Gpc3* (glypican-3)^38^, *Spp1* (osteopontin)^39^ and *Birc5* (survivin)^40^ in all DEN-treated mice compared to controls. However, at the protein level we found limited numbers of lesions immunopositive for AFP or GST-pi. This data suggests using either AFP or GST-pi as HCC markers in this model has limited utility.

Compared to previous popular HCC models, our DEN/TAA/HFD model is more representative of the contemporary clinic, where many patients have multiple causes of chronic liver injury that include overnutrition and dyslipidaemia. Gene expression patterns seen in human HCC indicate that there are diverse HCC types^26^, so a variety of approaches are necessary to recapitulate the human HCC portfolio in animals.

## Conclusion

Here we have described a novel model of HCC, achieved through the addition of a pro-steatotic insult to a pro-fibrotic insult in combination with a classical DEN model of chemically induced HCC. This approach reduces the time taken to reliably generate HCC from 32 to 24 weeks, and increases the variety of available models. This new model fits with the growing consensus that multiple ‘hits’ are required for severe NAFLD and HCC development. In summary, the addition of HFD to the DEN/TAA model increased HCC incidence, dysplastic lesion incidence, fibrosis, steatosis and inflammation at 24 weeks of age, which represents a useful model for the study of HCC development and therapy.

## Materials and Methods

### Mouse model

DEN (Sigma-Aldrich, St Louis, MO; catalogue number N0258–1G) was injected i.p. at 25 mg/kg body weight at 14 days of age into male C57BL/6J mice (see Fig. 1A). At 4 weeks of age, some mice were offered TAA (Alfa Aesar, Shanghai, China; catalogue number A12926) at 300 mg/L drinking water and some of those mice were offered HFD. The HFD was 45% kcal fat, 20% kcal protein, 35% kcal carbohydrate (Supporting information 1, 2). Mice were co-housed, with *ad libitum* food and water, filtered air and a 12 h light / dark cycle. These experiments were approved and monitored by Animal Ethics Committees of the University of Sydney and Area Health (Ethics protocols K75/5–2012/3/5754 and P013–017 ‘The biological roles of dipeptidyl peptidases’) and conducted in accordance with applicable laws and regulations.

### Histology

Tissue samples were formalin fixed then processed by the Histopathology Core Facility, Charles Perkins Centre, University of Sydney. H&E staining and immunohistochemistry (antibodies; Supporting information 3) on 5 µm paraffin sections have been described^41^.

Following 0.1% Picro-Sirius Red (Sigma-Aldrich, MO, catalogue number 365548) for 1 h at room temperature, slides were washed in acidified water containing 83% glacial acetic acid (Merck, Bayswater, Australia, catalogue number K48606863).

Bright-field imaging used a DM6000B microscope and Mosaic software (Leica, Wetzlar, Germany) to calculate % area of total tissue stained, at x200 magnification.

Steatosis, fibrosis, and inflammation was scored double blinded twice by trained researchers [authors JB, JH] from H&E slides, using a published scoring system for rodent NAFLD^42^. Lesions were identified by a trained pathologist [author JK] as either HCC, high grade dysplasia, low grade dysplasia, small cell change, focal fatty change or “other”.

### Flow cytometry

Liver tissue was incubated in Dulbecco’s modified Eagle’s medium (ThermoFisher Scientific, MA) 10% foetal calf serum (Scientifix, Cheltenham, Australia) 1 mg/ml collagenase (Sigma-Aldrich, catalogue number C5138) for 1 h, then leucocyte suspensions obtained and immunostained (antibodies; Supporting information 3) as described^43^. Cells were analysed on a custom 10-laser LSR II (BD Biosciences). Flow cytometric data were analysed with FlowJo software version 9.9 (TreeStar, Ashland, Ore)^43^.

### Real time quantitative PCR (qPCR)

Total liver RNA was isolated using PureLink RNA Mini kit (ThermoFisher, catalogue number 12183018 A), and quantified using the Qubit RNA BR Assay Kit (ThermoFisher Scientific, catalogue number Q10210) and a Qubit 3.0 Fluorimeter (ThermoFisher Scientific, catalogue number Q33216). The extracted RNA (1 μg) was reverse transcribed to cDNA using Superscript VILO cDNA synthesis kit (Invitrogen, catalogue number 11756050).

The expression of 87 genes was measured, in 5 livers per group, by qPCR using custom TaqMan array cards (format 384-well microfluidic card, Applied Biosystems, Foster City, CA), which were pre-spotted with custom designed dried-down TaqMan™ probes including 3 controls, *18S*, *Hprt* and *Rpl37a*, *as listed* (Supporting information 4). Real time qPCR used the QuantStudioTM 12 K Flex Real-Time PCR System (Applied Biosystems) and Expression Suite v1.0 (Applied Biosystems), utilizing the comparative Cτ (ΔΔCτ) method for data analyses. Gene expressions were normalized to the 3 endogenous controls.

### Data analysis

Data was analysed using a two-way analysis of variance (ANOVA) with Tukey’s multiple comparison test, Kruskal-Wallis comparison test, or one or two-tailed Mann Whitney U test, using GraphPad Prism (GraphPad v. 9.9, San Diego, CA, USA) and significance was assigned to p values; *0.05, **0.01, ***0.001, ****0.0001.

### Data availability statement

The datasets generated during the current study are available from the corresponding author upon reasonable request.

## Electronic supplementary material

Supplementary Information
