# Supplementary material for: Multiple liver insults synergize to accelerate experimental hepatocellular carcinoma
Source: Sci Rep. 2018 Jul 6;8:10283. doi: 10.1038/s41598-018-28486-8 (PMC6035229; doi:10.1038/s41598-018-28486-8)
Supplement: Supplementary file 1 — Supplementary Information [file 41598_2018_28486_MOESM1_ESM.pdf]

Multiple liver insults synergize to accelerate experimental hepatocellular carcinoma

James M Henderson[1,2], Natasa Polak[1,2], Jinbiao Chen[1,2], Ben Roediger[1,2], Wolfgang Weninger[1,2,3], James Kench[2,3], Geoffrey W McCaughan[1,2,3], Hui Emma Zhang[1,2], Mark D Gorrell[1,2,4]

1. Centenary Institute, Locked Bag No.6, Newtown, New South Wales, 2042, Australia.
2. The University of Sydney School of Medicine, New South Wales, 2006, Australia.
3. Royal Prince Alfred Hospital, Camperdown, New South Wales, 2050, Australia.
4. Charles Perkins Centre, The University of Sydney, Australia.

Correspondence

Mark D. Gorrell,

Liver Enzymes in Metabolism and Inflammation Program,

Centenary Institute of Cancer Medicine and Cell Biology

Locked Bag No. 6, Newtown, NSW 2042, Australia

Tel: 61-2-95656156; Fax: 61-2-95656101.

E-Mail address: m.gorrell@centenary.usyd.edu.au

Supporting information 1: High fat diet (HFD) recipe. The HFD was prepared in-house and based on rodent diet no. D12451 (Research Diets New Brunswick, USA).

Ingredients

- 261 g of Casein (Sigma cat no. C7078)
- 224 g of Sucrose (MP Biomedical cat no. 904713)
- 214.8 g of Starch (MP biomedical 102955)
- 51 g of AIN mineral mix (MP biomedical cat no. 905455)
- 57 g of Bran (homebrand Natural Bran)
- 3.4 g of Methionine (Sigma-Aldrich cat no. M9625)
- 23 g of Gelatine (Wards, Altona, Australia)
- 4.6 g of choline bitartrate (Sigma cat no.C1629)
- 14.8 g of AIN vitamins (MP biomedical cat no.960098)
- 6 g of Cholesterol (Sigma Cat no.8503)
- 250 g of room temperature Lard (Yorkfoods natural lard) (Goulburn, Australia)
- 35 ml of safflower oil (Melrose organic Safflower oil) (Mount Waverly, Australia)
- 2 drops of strawberry flavouring (Queen strawberry essence (Alderley, Australia).

[Regular food items are supermarket – sourced]

Method

Remove Lard from 4 ^{0}C and bring to room temperature. In a clean container add Casein, Sucrose, Starch, AIN mineral mix, Bran, Methionine, Gelatine, choline bitartrate, AIN vitamins, Cholesterol and mix through thoroughly. Add room temperature Lard and safflower oil and 2 drops of strawberry flavouring. Mix together until dough - like texture, then store under nitrogen in 4 ^{0}C for up to two weeks. Local animal facility sources these items: Mice are housed on Pure-o Cel® bedding (Anderson lab bedding, Ohio, USA) and one wooden chew block (Able Scientific cat no. ASAEC-A) per cage is provided as the chew blocks, extra vitamins and non-edible bedding are essential. The extra vitamins were provided as soluble berry flavoured multivitamins (Berocca, Germany; 1 tablet / L). The drinking water was changed twice per week; once with multivitamins, once without.

Note

Whenever HFD is used, in vivo quality control is done, which is 2 cages of mice running at all times, with weight gain charted weekly **(Supporting information 2)**. These are an additional cage of mice treated only with that diet, versus a chow control cage of mice. This is quality control on the diet. Whenever HFD-induced weight gain ceases in these controls the diet and housing conditions are re-evaluated to solve the problem. These mice run for 20 weeks of HFD, then 2 new cages are set up.

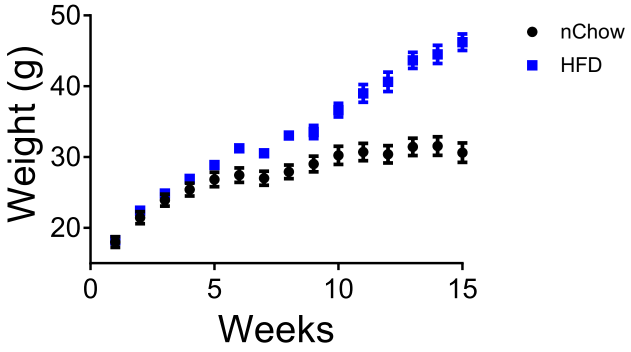

Supporting information 2: Diet quality was checked by monitoring weight gain of mice fed *ad libitum* with either the HFD or normal chow (nChow) (n=3-5, Mean and SEM).

Supporting information 3: Antibodies

Antibody	Isotype	Supplier	Catalogue No.	Working dilution
Alpha-SMA	Rabbit	Abcam	Ab32575	1:100
Dipeptidyl peptidase 9	Rabbit	Abcam	Ab42080	1:100
F4/80	Rat	Dr Bertolino	Hybridoma A3-1	neat
Glutathione S- transferase Pi	Rabbit	Santa Cruz	Sc-134469	1:100
Alpha-fetoprotein	Goat	R&D systems	AF5369	1:100
Anti-goat-HRP	Rabbit	Dako	P0449	1:100
Anti-rat-HRP	Goat	Invitrogen	#31470	1:100
Anti-Rabbit-HRP	Goat	Dako	P0448	1:100
CD45 BUV395	Rat	BD Horizon	564279	1:100
CD11b BV421	Rat	BD Horizon	562605	1:100
F4/80 PE-cy7	Rat	Bio Legend	123114	1:100
Ly6C PercP-cy5.5	Rat	Bio Legend	128012	1:100
CD19 PE	Rat	BD Horizon	561736	1:100
Ly6G Alexa647	Rat	Bio Legend	127610	1:100
CD90.2 FITC	Rat	BD Pharmingen	553004	1:100

Supporting information 4: List of gene names and probes.

Gene symbol	gene name	gene alias	Primer/probe assay	Gene function	Amplicon length
RN18S (human)	eukaryotic 18S rRNA	18S RNA	Hs99999901_s1	House keeping	187
Afp	alpha fetoprotein		Mm00431715_m1	HCC marker	96
Bax	BCL2-associated X protein		Mm00432051_m1	Apoptosis	84
Bcl2a1a	B cell leukemia/lymphoma 2 related protein A1a	A1, BB218357, Bcl2a1, Bfl-1, Hbpa1	Mm03646861_mH	Apoptosis	112
Bcl2l1	BCL2-like 1	Bcl(X)L, Bcl-XL, Bcl2l, BclX, bcl-x, bcl2-L-1	Mm00437783_m1	Apoptosis	65
Birc2	baculoviral IAP repeat-containing 2	AW146227, Api1, Api2, Birc3, HIAP1, HIAP2, IAP1, IAP2, MIAP1, MIAP2, MIHB, MIHC, RNF48, cIAP1, cIAP2, mcIAP1	Mm00431811_m1	NF□b target. Apoptosis.	90
Birc3	baculoviral IAP repeat-containing 3	AW107670, Api1, Api2, Birc2, HIAP2, IAP1, IAP2, MIAP1, MIAP2, MIHB, MIHC, RNF49, cIAP-1, cIAP-2	Mm01168413_m1	NF□b target. Apoptosis.	60
Birc5	baculoviral IAP repeat-containing 5	AAC-11, Api4, TIAP, survivin40	Mm00599749_m1	HCC marker	83
Braf	Braf transforming gene	9930012E13Rik, AA120551, AA387315, AA473386, B-raf, Braf-2, Braf2, C230098H17, C87398, D6Ertd631e	Mm01165837_m1	Oncogene	94
Casp1	caspase 1	ICE, Il1bc	Mm00438023_m1	Apoptosis/pyroptosis	99
Ccl2	chemokine (C-C motif) ligand 2	AI323594, HC11, JE, MCAF, MCP-1, MCP1, SMC-CF, Scya2, Sigje	Mm00441242_m1	Chemokine	74
Ccl5	chemokine (C-C motif) ligand 5	MuRantes, RANTES, SISd, Scya5, TCP228	Mm01302427_m1	Chemokine	103
Ccnd1	cyclin D1	AI327039, CycD1, Cyl-1, PRAD1, bcl-1, cD1	Mm00432359_m1	Cancer	58
Ccne1	cyclin E1	AW538188, CycE1	Mm01266311_m1	Cancer	148
Ccr2	chemokine (C-C motif) receptor 2	Cc-ckr-2, Ccr2a, Ccr2b, Ckr2, Ckr2a, Ckr2b, Cmkbr2, mJe-r	Mm00438270_m1	Chemokine	100
Cd163	CD163 antigen	CD163v2, CD163v3	Mm00474091_m1	Macrophage	83
CD21	complement receptor 2	C3DR, Cr2, CD35, Cr-1, Cr-2, Cr1	Mm00801681_m1	Immune system	77
Cd36	CD36 antigen	FAT, GPIV, Scarb3	Mm01135198_m1	Fatty acid transport	112
Cd47	CD47 antigen (Rh-related antigen, integrin-associated signal transducer)	9130415E20Rik, AA407862, AI848868, AW108519, B430305P08Rik, IAP, Itgp	Mm00495011_m1	Macrophages	77
Cd64	Fc receptor, IgG, high affinity I	AI323638, AV092959, Fcgr1, FcgammaRI, IGGHAFC	Mm00438874_m1	Macrophages	58
Cd68	CD68 antigen	Lamp4, Scard1, gp110	Mm03047343_m1	Macrophages	109
Cdk1	cyclin-dependent kinase 1	Cdc2, Cdc2a, p34<CDC2>	Mm00772472_m1	Senescence	76
Cdk4	cyclin-dependent kinase 4	Crk3	Mm00726334_s1	Cancer kinase	54
Cdk9	cyclin-dependent kinase 9 (CDC2-related kinase)	PITALRE	Mm01731275_m1	Cancer kinase	150

Supporting information 4 (continued): List of gene names and probes.

Gene symbol	gene name	gene alias	Primer/probe assay	Gene function	Amplicon length
Cdkn2a	cyclin-dependent kinase inhibitor 2A	ARF-INK4a, Arf, INK4a-ARF, Ink4a/Arf, MTS1, Pctr1, p16, p16(INK4a), p16INK4a, p19ARF	Mm00494449_m1	Senescence	55
Col1a2	collagen, type I, alpha 2	AA960264, AI325291, Col1a-2, Cola-2, Cola2, oim	Mm00483888_m1	Extracellular matrix (ECM)	67
Ctgf	connective tissue growth factor	Ccn2, Fisp12, Hcs24, fisp-12	Mm01192933_g1	Growth factor for ECM	67
Cx3cr1	chemokine (C-X3-C motif) receptor 1		Mm00438354_m1	Chemokine	70
Cxcr3	chemokine (C-X-C motif) receptor 3	Cd183, Cmkar3	Mm00438259_m1	Chemokine/Interleukins	58
Foxp3	forkhead box P3	JM2, scurfin, sf	Mm00475162_m1	Immune system	74
Gpc3	glypican 3	OCI-5	Mm00516722_m1	HCC marker. DPP4 ligand	91
Hif1	hypoxia inducible factor 1, alpha subunit	AA959795, HIF1alpha, MOP1, bHLHe78	Mm00468869_m1	Oxidative stress	75
Hprt1	hypoxanthine guanine phosphoribosyl transferase		Mm00446968_m1	House keeping gene	65
Hras	Harvey rat sarcoma virus oncogene	H-ras, Ha-ras, Harvey-ras, Hras-1, Hras1, Kras2, c-H-ras, c-Ha-ras, c-rasHa, ras	Mm01275932_g1	Oncogene	86
Itgax	integrin alpha X	AI449405, Cd11c, Cr4, N418	Mm00498698_m1	Integrin. ECM binding	96
Keap1	kelch-like ECH-associated protein 1	INRF2, mKIAA0132	Mm00497268_m1	Macrophage	71
Klrk1	killer cell lectin-like receptor subfamily K, member 1	D6H12S2489E, NKG2-D, Nkg2d	Mm00473603_m1	Immune system	124
Kras	v-Ki-ras2 Kirsten rat sarcoma viral oncogene homolog	AI929937, K-ras, Ki-ras, Kras-2, Kras2, p21B, ras	Mm00517492_m1	Proto-oncogene, GTPase	125
Loxl2	lysyl oxidase-like 2	1110004B06Rik, 4930526G11Rik, 9430067E15Rik, LOX-L2	Mm00804740_m1	Collagen crosslinking	82
Mif	macrophage migration inhibitory factor	GIF, Glif	Mm01611157_gH	Growth factor	177
Myc	myelocytomatosis oncogene	AU016757, Myc2, Niard, Nird, bHLHe39	Mm00487804_m1	Oncogene	89
Nfkbia	nuclear factor of kappa light polypeptide gene enhancer in B cells inhibitor, alpha	AI462015, Nfkbi	Mm00477798_m1	Immune system	70
Nfkbib	nuclear factor of kappa light polypeptide gene enhancer in B cells inhibitor, beta	I(Kappa)B(beta), I-kappa-B-beta, IKB-beta, IKappaBbeta, IkB, IkBb, NF-kappa-BIB, ikB-B	Mm00456853_m1	Immune system	64
Nkap	NFKB activating protein	2610020O08Rik, AA987160, AI849147, AL024236	Mm00482418_m1	Immune system	87
Nqo1	NAD(P)H dehydrogenase, quinone 1	AV001255, Dia4, Dtd, Nmo-1, Nmo1, Nmor1, Ox-1, Ox1, Qr1	Mm01253561_m1	NAD(P)H dehydrogenase, quinone 1	81
Pai1	serine (or cysteine) peptidase inhibitor, clade E, member 1	PAI-1, PAI1, Planh1, serpine1	Mm00435858_m1	ECM angiogenesis	87
Pbx1	pre B cell leukemia homeobox 1	2310056B04Rik, D230003C07Rik, Pbx-1	Mm04207617_m1	Stellate cell	91
Pdk4	pyruvate dehydrogenase kinase, isoenzyme 4	AV005916	Mm01166879_m1	Cancer kinase	66
Pdl1	CD274 antigen	A530045L16Rik, B7h1, Pdcd1l1, Pdcd1lg1, Pdl1	Mm00452054_m1	Programmed death and apoptosis	77
Pten	phosphatase and tensin homolog	2310035O07Rik, A130070J02Rik, AI463227, B430203M17Rik, MMAC1, TEP1	Mm00477208_m1	A tumor suppressor	73
Rpl37a	ribosomal protein L37a		Mm01546394_s1	House keeping gene	111
Spp1	secreted phosphoprotein 1	Osteopontin, 2AR, Apl-1, BNSP, BSPI, Bsp, ETA-1, Eta, OP, Opn, Opnl, Ric, Spp-1	Mm00436767_m1	Immune system	114
Tgfb1	transforming growth factor, beta 1	TGF-beta1, TGFbeta1, Tgfb, Tgfb-1	Mm01178820_m1	Oncogene	59
Tnf	tumor necrosis factor	DIF, TNF-a, TNF-alpha, TNFSF2, TNFalpha, Tnfa, Tnfsf1a	Mm00443258_m1	Immune system	81
Trp53	transformation related protein 53	Tp53, bbl, bfy, bhy, p44, p53	Mm01731290_g1	Tumor protein p53	119
Xiap	X-linked inhibitor of apoptosis	1110015C02Rik, Aipa, Api3, Birc4, IAP3, ILP-1, MIHA	Mm01311594_mH	NF□b target gene. Apoptosis	68

Gene symbol	gene name	gene alias	Primer/probe assay	Gene function	Amplicon length
Arg1	arginase, liver	AI, AI256583, Arg-1, PGIF	Mm00475988_m1	Macrophage	65
Bambi	BMP and activin membrane-bound inhibitor	2610003H06Rik	Mm03024088_g1	TGFb signalling	127
Bcl2	BCL2-like 11 (apoptosis facilitator)	1500006F24Rik, Bim, Bod, bcl2-L-11	Mm00437796_m1	Apoptosis	64
Bhlhe40	basic helix-loop-helix family, member e40	Bhlhb2, C130042M06Rik, CR8, Clast5, Dec1, Sharp2, Stra13, Stra14	Mm00478593_m1	Senescence	58
Casp3	caspase 3	A830040C14Rik, AC-3, Apopain, CASP-3, CC3, CPP-32, CPP32, Caspase-3, Lice, SCA-1, Yama, mldy	Mm01195085_m1	Apoptosis	70
Casp9	caspase 9	AI115399, APAF-3, AW493809, CASP-9, Caspase-9, ICE-LAP6, Mch6	Mm00516563_m1	Apoptosis	68
Ccl21	chemokine (C-C motif) ligand 21A (serine)	6CKBAC2, 6Ckine, ALP, AW987545, CKb9, SCYA21a, SLC, Scya21, Scya21b, Tca4, plt	Mm03646971_gH	Chemokine	91
Cdkn2b	cyclin-dependent kinase inhibitor 2B (p15, inhibits CDK4)	AV083695, INK4b, MTS2, p15, p15(INK4b), p15INK4b	Mm00483241_m1	Senescence	112
Cxcl10	chemokine (C-X-C motif) ligand 10	C7, CRG-2, INP10, IP-10, IP10, Ifi10, Scyb10, gIP-10, mob-1	Mm00445235_m1	Chemokine	59
Cxcr2	chemokine (C-X-C motif) receptor 2	CD128, CDw128, Cmkar2, Gpcr16, IL-8Rh, IL-8rb, IL8RA, Il8rb, mIL-8RH	Mm00438258_m1	Chemokine	67
Cxcr4	chemokine (C-X-C motif) receptor 4	CD184, Cmkar4, LESTR, PB-CKR, PBSF/SDF-1, Sdf1r, b2b220Clo	Mm01292123_m1	Chemokine	99
Egf	epidermal growth factor	AI790464	Mm01316968_m1	Growth factor	64
Egfr	epidermal growth factor receptor	9030024J15Rik, AI552599, Erbb, Errb1, Errp, Wa5, wa-2, wa2	Mm01187858_m1	Growth factor	101
Foxo1	forkhead box O1	AI876417, Afxh, FKHR, Fkhr1, Foxo1a	Mm00490671_m1	Metabolism	86
G6pc	glucose-6-phosphatase, catalytic	AW107337, G6Pase, G6pt, Glc-6-Pase	Mm00839363_m1	Metabolism	116
Gck	glucokinase	GLK, Gk, Gls006, HK4, HKIV, HXKP, Hlb62, MODY2	Mm00439129_m1	Metabolism	69
Hgf	hepatocyte growth factor	C230052L06Rik, HGF/SF, NK1, NK2, SF, SF/HGF	Mm01135184_m1	Growth factor	85
Hnf4a	hepatic nuclear factor 4, alpha	HNF-4, Hnf4, Hnf4alpha, MODY1, Nr2a1, Tcf14	Mm00433964_m1	Growth factor	114
Igf1	insulin-like growth factor 1	C730016P09Rik, Igf-1, Igf-I	Mm00439560_m1	Growth factor	77
Il1b	interleukin 1 beta	IL-1beta, Il-1b	Mm00434228_m1	Immune system	90
Il2ra	interleukin 2 receptor, alpha chain	CD25, Il2r, Ly-43	Mm01340213_m1	Immune system	83
Il6	interleukin 6	Il-6	Mm00446190_m1	Chemokine. Growth	78
IL8	chemokine (C-X-C motif) ligand 8	Il8, Scyb15, lungkine, weche	Mm00441263_m1	Chemokine	66
mTor	mechanistic target of rapamycin (serine/threonine kinase)	2610315D21Rik, AI327068, FRAP, FRAP2, Frap1, RAFT1, RAPT1, flat	Mm00444968_m1	Metabolism	65
Nk	killer cell lectin-like receptor subfamily B member 1C	AI462337, CD161, Ly-59, Ly55c, Ly59, NK-RP1, NK1.1, NKR-P1.9, NKRP1, NKRP140, Nk-1, Nk-1.2, Nk1, Nk1.2, Nkrp1c, ly-55c, Klrb1c	Mm00824341_m1	Immune system	145
NKT	CD1d2 antigen	Cd1d2, CD1.2, Cd1b, Ly-38	Mm00776138_gH	Immune system	120
Nos2	nitric oxide synthase 2, inducible	NOS-II, Nos-2, Nos2a, i-NOS, iNOS	Mm00440502_m1	Oxidative stress. Macrophage	66
Pck1	phosphoenolpyruvate carboxykinase 1, cytosolic	AI265463, PEPCK, Pck-1	Mm01247058_m1	Cancer kinase	61
Pd1	programmed cell death 1	Pdcd1,Ly101, PD-1, Pdc1	Mm01285676_m1	Apoptosis	64
Pgf	placental growth factor	AI854365, PIGF, Plgf	Mm00435613_m1	Growth factor	75
Smad4	SMAD family member 4	AW743858, D18Wsu70e, DPC4, Madh4	Mm03023996_m1	TGF-beta signaling	71
Stat3	signal transducer and activator of transcription 3	1110034C02Rik, AW109958, Aprf	Mm01219775_m1	Signaling	75
Vegf	vascular endothelial growth factor A	Vegf, Vpf, Vegfa	Mm00437306_m1	Growth factor	61

Supporting information 4 (continued): List of gene names and probes.

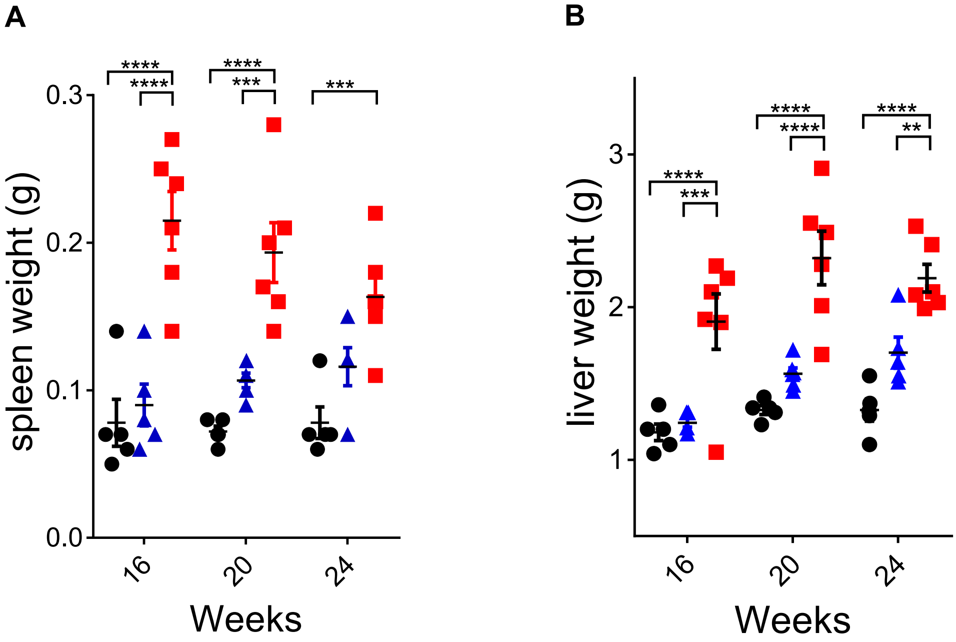

Supporting information 5: Spleen weights **(A)** and liver weight **(B)**. n=5-6. Mean and SEM. Statistical significance used Kruskal-Wallis test: **p<0.01, **p<0.005, ***p<0.001, ****p<0.0005.*

Supporting information 6: Observed lesions in H&E stained liver at 24 weeks of age

Treatment	HCC	High grade dysplasia	Low grade dysplasia	Small cell change	Focal fatty change
Control	0	0	0	0	0
Control	0	0	0	0	0
Control	0	0	0	0	0
Control	0	0	0	0	0
Control	0	0	0	0	0
DEN/TAA	0	0	0	1	0
DEN/TAA	0	0	2	1	0
DEN/TAA	0	5	3	2	0
DEN/TAA	0	1	2	1	0
DEN/TAA	1	1	0	3	0
DEN/TAA/HFD	3	2	1	0	0
DEN/TAA/HFD	1	1	1	0	3
DEN/TAA/HFD	1	0	2	0	0
DEN/TAA/HFD	0	3	2	0	0
DEN/TAA/HFD	3	5	1	0	0
DEN/TAA/HFD	1	1	4	0	0

HCC, dysplasia, small cell change, and focal fatty change are illustrated in **Figure 3**.
